# Horizontal gaze palsy with progressive scoliosis: Three novel *ROBO3* mutations and descriptions of the phenotypes of four patients

**Published:** 2011-07-20

**Authors:** Alexander E. Volk, Oliver Carter, Julia Fricke, Peter Herkenrath, Jörg Poggenborg, Guntram Borck, Andre W. Demant, Roland Ivo, Peer Eysel, Christian Kubisch, Antje Neugebauer

**Affiliations:** 1 Institute of Human Genetics, University Hospital of Cologne, Cologne, Germany; 2 Institute for Genetics, University of Cologne, Cologne, Germany; 3 Center for Molecular Medicine Cologne, University of Cologne, Cologne, Germany; 4 Institute of Human Genetics, University Hospital of Ulm, Ulm, Germany; 5 Department of Ophthalmology, University Hospital of Cologne, Cologne, Germany; 6 Department of Pediatrics, University Hospital of Cologne, Cologne, Germany; 7 Department of Radiology, University Hospital of Cologne, Cologne, Germany; 8 Department of Orthopedic and Trauma Surgery, University Hospital of Cologne, Cologne, Germany

## Abstract

**Purpose:**

Clinical and molecular characterization of patients with horizontal gaze palsy with progressive scoliosis (HGPPS) to extend existing knowledge of the phenotype caused by mutations in the Roundabout homolog of Drosophila 3 (*ROBO3)* gene.

**Methods:**

Four patients (aged 6 months to 13 years), two of them siblings, with features of horizontal gaze palsy and their parents were examined clinically and by molecular testing of the *ROBO3* gene. The three families were unrelated, but parents in each family were consanguineous.

**Results:**

We identified three novel homozygous *ROBO3* mutations in four patients with typical ophthalmologic signs of HGPPS. We found an exonic insertion/deletion mutation (c.913delAinsTGC; p.Ile305CysfsX13), a 31 bp deletion including the donor splice site of exon 17 and adjacent exonic and intronic sequences (c.2769_2779del11, 2779+1_+20del20), and a missense mutation located next to a splice donor site (c.3319A>C) resulting in skipping of exon 22, as shown by cDNA analysis.

**Conclusions:**

We describe three novel mutations in the *ROBO3* gene and the detailed clinical phenotype of HGPPS. One patient displayed marked convergence upon attempting smooth pursuits to both sides. In one patient, the typical ophthalmologic phenotype, the neuroradiologic findings, and molecular testing led to the diagnosis even before scoliosis developed. In addition to the typical magnetic resonance imaging brain signs of HGPPS, this patient had marked hypoplasia of the frontal lobes and corpus callosum. In summary, diagnosis of HGPPS may be established by ophthalmologic and molecular investigation early in life, allowing ongoing orthopedic surveillance from an early stage.

## Introduction

Bi-allelic mutations in the Roundabout homolog of Drosophila 3 (*ROBO3*) gene cause horizontal gaze palsy with progressive scoliosis (HGPPS; OMIM 607313), a rare autosomal recessive disorder with oculomotor and general disturbances in innervation [1,2]. The *ROBO3* gene encompasses 28 exons and encodes a protein of 1,386 amino acids. Homologous to other members of the Roundabout gene family, *ROBO3* contains a putative extracellular segment with five immunoglobulin-like motifs and three fibronectin-like motifs, a transmembrane segment, and an intracellular segment with three cytoplasmic signaling motifs [1]. To date, 24 distinct mutations located in different domains of the *ROBO3* gene have been described [1–6]. Mutations in *ROBO3* are associated with noncrossing of selected axonal paths in the central nervous system that are normally subjected to midline crossing during embryonic development.

The oculomotor signs of HGPPS comprise congenital absence of horizontal gaze under the conditions of conjugate gaze at smooth pursuit, saccades, and vestibulo-ocular or optokinetic responses. In the majority of cases, adduction is preserved under the condition of convergence. Occasionally, patients were reported to use convergence in side gaze to further move the adducted and fixating eye over the midline [2,7]. One patient displayed bilateral synergistic convergence without pupil constriction upon attempting to gaze horizontally to one side or the other [5]. Vertical eye movements are mainly unaffected [2,7]. Nystagmus presents in many patients and is mostly horizontal and pendular, with low amplitude [2,3,8]. Sporadically, nystagmus is accompanied by slight involuntary head movements. Several patients display asynchronous blinking [5,7]. Visual fields, pupil function, accommodation, and anterior and posterior segments of the eye are unremarkable. Visual acuity is reported not to be grossly impaired [4].

Scoliosis in HGPPS is mostly diagnosed after the age of two years and progresses within the first decade. Treatment options are physiotherapy, three-dimensional correction using thermoplastic braces, and corrective spine surgery [7].

At first glance, patients seem neurologically unimpaired. However, motor development is delayed in many patients [2,5,7]. Cognitive function is not grossly impaired, yet some patients perform with a lower mean average score on general intelligence tests [7,9].

Conventional magnetic resonance imaging (MRI) typically reveals a characteristic brainstem configuration with anterior and posterior midline clefts. The unusual anterior cleft at the medullary level is accompanied by an anteriorly flattened medulla with a butterfly-like bifid appearance in axial sections. The pontine tegmentum is flattened and the facial colliculi do not protrude markedly into the fourth ventricle. The anterior-posterior diameter of both the pons and the medulla is reduced, and the cerebellar peduncles are described as being reduced in some reports [5,10]. The supratentorial structures, specifically the corpus callosum and the optic chiasm, appear normal [7,9]. A single child has been reported with prominent cerebral fluid spaces anterior to the frontal lobes. Additionally, widened sylvian fissures exist in this case, “suggesting that the accompanying macrocephaly resulted from benign external hydrocephalus” [2]. The extraocular muscles [1,5,7] and the third and sixth cranial nerves [1,5] appear normal. Diffusion tensor imaging shows a reduction of crossing fibers in the brainstem [8,10]. Lack of decussation of the cerebellar peduncles and absence of normal pyramidal tract crossing is seen, whereas the corpus callosum has normal fiber tract orientation. Functional MRI with paradigms for somatosensory and motor-evoked potentials shows responses dominantly ipsilateral to the stimulation side [8]. Electrophysiologic examination in individual patients reveals functional evidence for at least partial noncrossing of the pyramidal tract and the lemniscal pathway [1,9].

To broaden the existing knowledge of the clinical phenotypes and genotypes of this rare disease, we comprehensively examined four patients suspected of having HGPPS both clinically and by molecular investigation of *ROBO3*.

## Methods

### Clinical data

The four patients we referred to the outpatient care of the Department of Ophthalmology due to eye motility disorders. As HGPPS was suspected in these patients they underwent detailed ophthalmologic and orthoptic examinations, including anterior and posterior segments, ocular motility, ocular alignment, and tests for binocular vision. Complementary neuropediatric, neuroradiologic, and orthopedic examinations were conducted when necessary.

### Molecular investigations

Genomic DNA was extracted from a blood sample of each participant. The entire coding and flanking intronic sequences of the *ROBO3* gene were amplified by PCR and sequenced. In one out of two siblings harboring a nucleotide exchange next to the splice-donor site and in their father, RNA from whole blood was isolated for further analysis using the PAXgene Blood RNA Kit (Qiagen, Hilden, Germany). The cDNA was synthesized using the Qiagen® OneStep reverse transcription (RT)-PCR kit according to manufacturer’s instructions. Primer sequences are listed in Table 1. For PCR, a touchdown protocol (65 °C–55 °C) was used. The sequences were compared with the *ROBO3* reference sequence (GenBank NM_022370). If a potentially disease-causing variant was detected, the PCR amplification and sequencing reaction were repeated, and at least 144 healthy controls of Caucasian (n=86) and Turkish (n=58) ethnicity were screened to exclude common polymorphisms. For splice prediction, the program NNSplice was used. All participating families gave their written informed consent. The study was approved by the local Ethics Committee of the Medical Faculty, University of Cologne, Germany.

**Table 1:** Sequences of oligonucleotide primers used in this study and PCR product size.

**Fragment name**	**Forward primer(5′-3′)**	**Reverse primer(5′-3′)**	**Product size (bp)**
Exon 1	ACGAAGAGGCACCGACCGTAC	CACTCATGTGCTCGAAGCTCC	498
Exon 2	GCAGAGAAGCATAGGAGATGG	CCAAAGAAGGATGCTGAGTGG	559
Exon 3	TGGGAACGAATTCCAGTCTGC	AGTCTCTGGTTAGCTAATGCC	381
Exon 4/5	GTCTCCTTTATCAGCTTGCTGTG	TGGGGACTGGATAGGACTGGG	632
Exon 6	ATGGGTAACCAGCTTCTGTCC	TGCAGAAGTCTCAGGTGTTCC	385
Exon 7	GGAAGAGATGGATATCTGCTCC	CTCTAAGAACCTACCATCCAGG	503
Exon 8	CATCACTGCTGGAGACAGACG	ATTAGTGGCTTCTGCTGCTGG	466
Exon 9/10	CTGATCACCGGAAGTTTCAGG	TCCTCACCACCGTAACCATGG	759
Exon 11	TAGCCCACTCTGACCATCACC	CCTGCCTACTCCATCTCATCC	443
Exon 12	GATTCTCCAGTACCCTCTTGC	GTGAGAGGCAAACATGATCTCC	453
Exon 13/14	GTGCTCTGGAGTACTAAGTGG	TAGGGAACTGAGAGCCATTCC	792
Exon 15	AATTTGCAGGAGGAGAGCAGG	GCTCCAAATGGTAGAGAAGCC	477
Exon 16/17	GAGAAGAGAGAGGTTTCCAAGTG	AAGAGGCTGAACTCCAGGTGC	779
Exon 18/19	GTCTCTGGAGAGATTCTGAGG	TTGGGGTGGGAGGTGGCCATG	513
Exon 20	CCGACAGGCCACTCTTCTTCC	CATCCTAAACCAGGGTGGAGG	477
Exon 21	CTCTCTGAGGTTGGTCAGTCC	GAGATGAGACAGGTCAGAAGG	505
Exon 22/23	CCCAAAGAGGATCCTCCTCCC	CACTTCCACCCTGACACAGCC	756
Exon 24/25	GCTCTCCTTATGCTTCTCTAGC	TCACCTGCATTCCCTGGCACC	694
Exon 26	GGGATAGGCAGGAAACCAAGG	CTCAGAGAGTTAGAGAGACAGG	483
Exon 27	ATGTGCCGGTCACTCTGCTGC	GTCACTTCTACAGGATTCCTCC	512
Exon 28	CACATAATAGAGGAGCCTGGG	CACAGAACCAGACAGACAGGG	385
cDNA22/24	TCTCCCTGTATCTAGCTCAGAC	ATACACTGAGAGGGGAACTCG	576 (cDNA)

## Results

### Clinical findings in patient 1

**Figure 1:**
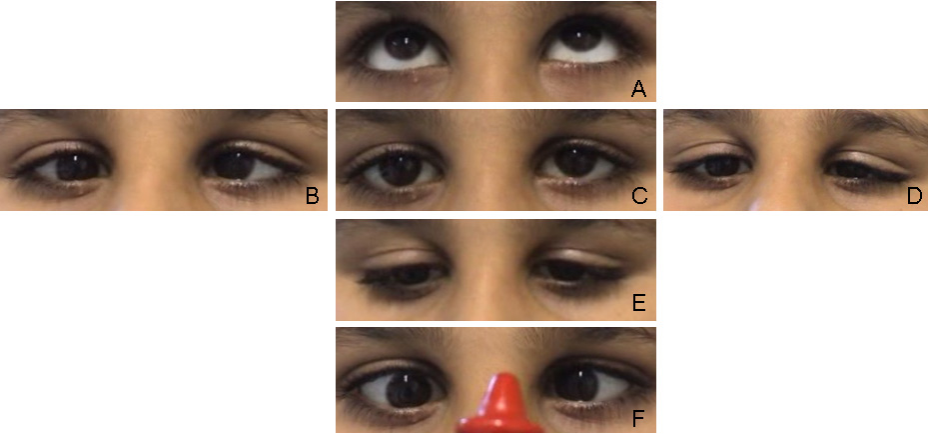
Patient 1 at the age of 8 years, after a bilateral medial rectus recession at the age of 2.5 years. **A**-**F** show the eye position at different attempted movements. The eye position at upgaze is shown in **A**. **B** depicts the eye position at right gaze. **C** shows the eyes in primary position. The eye position in left gaze, downgaze and under the condition of convergence is demonstrated in **D**, **E**, **F**, respectively.

**Figure 2:**
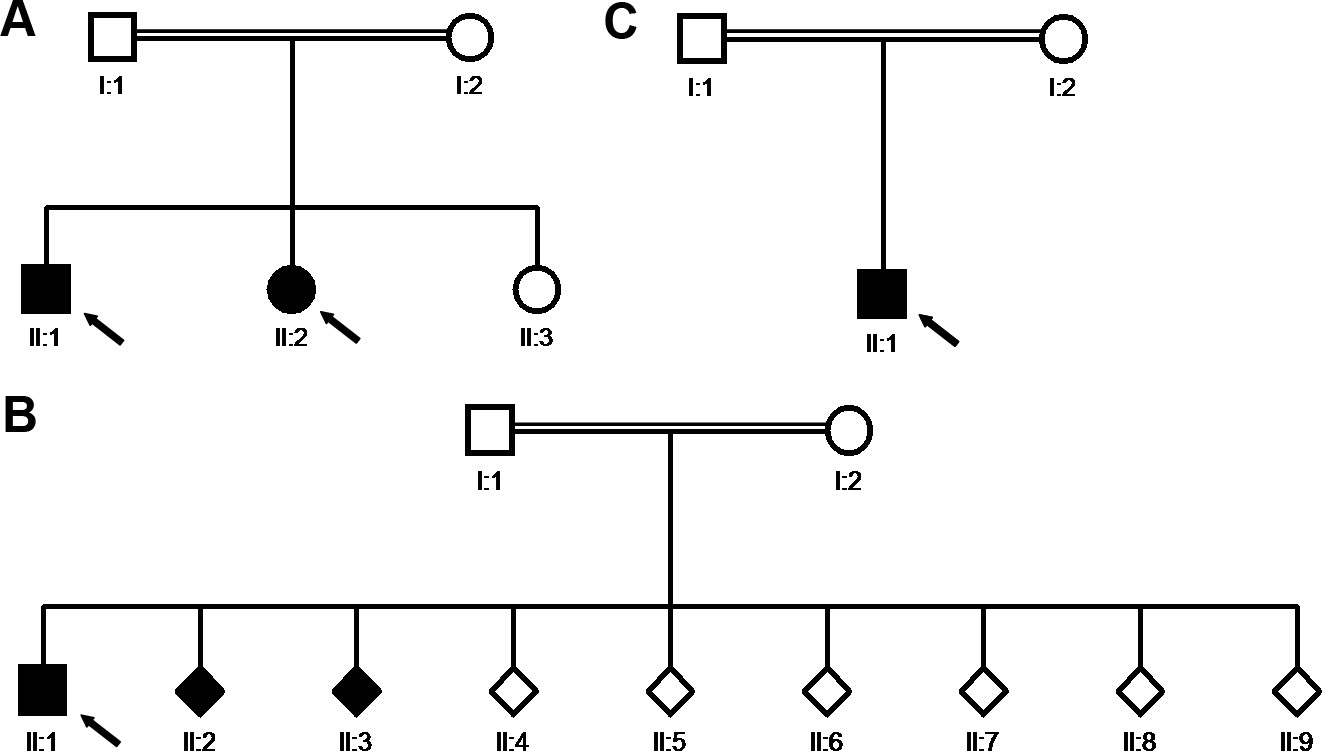
Pedigree diagrams of the three participating families. Circles represent females, squares males and rhombi offspring of unknown sex. Filled symbols indicate clinically affected individuals. HGPPS-patients are marked by arrows: patient 1 (II.1) and patient 2 (II.2) in **A**, patient 3 (II.1) in **B** and patient 4 (II.1) in **C**. All patients were born to consanguineous parents.

The 12-year-old boy displayed limited abduction and adduction in both eyes. He had a history of convergent strabismus, treated elsewhere. The convergent angle was 7° at 4 months of age and 20° at 2.5 years of age. With the diagnosis of bilateral Duane’s syndrome, a bilateral medial rectus recession of 6 mm was performed. Figure 1 shows his eyes at the age of 8 years under different gaze conditions. He presented with microhypertropia of the left eye with fusion under Bagolini’s striated glasses. Abduction could not be elicited by smooth pursuits, saccades, or optokinetic reflex. Adduction was seen on the attempted convergence at near fixation. To follow near objects, adduction seemed to be obtained by an effort to converge. Occasionally, his left lid fissure narrowed at attempted left gaze at near fixation. Vertical movements were unimpaired. He had a fine, horizontal pendular nystagmus with intermittent head-nodding and moderate anisohyperopia. Corrected visual acuity was 0.8 in the right eye and 0.4 in the left amblyopic eye. Cranial MRI showed a flattened pons. Thoracolumbar scoliosis was diagnosed at the age of 6 years and had been progressive. Current radiographs displayed a Cobb angle of 32°, and treatment consisted of physiotherapy and corrective brace therapy. Motor development was slightly delayed, because he started walking at about 1.5 to 2 years of age. The patient’s parents were of Turkish origin and were first cousins, healthy, and without any signs of ocular motility disturbance. A younger sister was also affected (patient 2), but the third sibling had normal eye movements and no signs of scoliosis (Figure 2A).

### Clinical findings in patient 2

**Figure 3:**
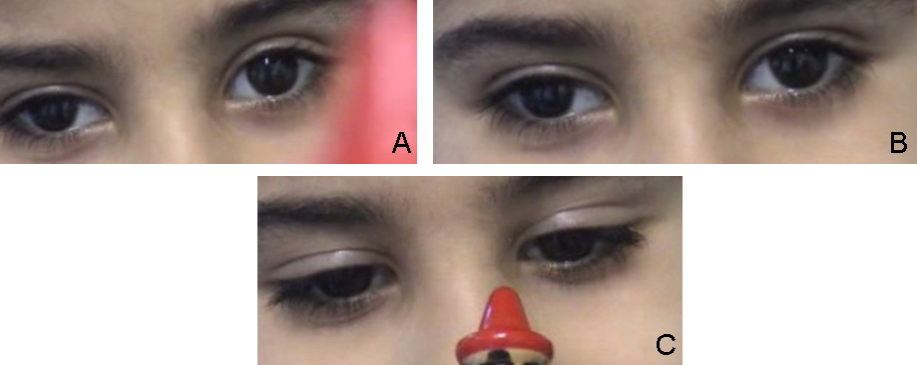
Patient 2 at the age of six years. Eye position at primary gaze, at attempted left gaze, and under the condition of convergence is shown in **A**, **B**, and **C**, respectively.

The nine-year-old sister of patient 1 presented with −12° of exotropia and left microhypertropia. No strabismus surgery had so far been performed. At the age of 2.5 years, her eye position was documented elsewhere to be +0.5° of esotropia, with binocular vision. Minor adducting eye movements could be elicited under the conditions of accommodation to a near target. A fine, dissociated pendular and slightly rotational nystagmus was observed. Beyond these movements, no larger horizontal excursions of the eyes existed. Vertical movements were normal. Corrected visual acuity was 0.6 in each eye. Figure 3 shows her eye movements at the age of 6 years. The MRI showed a flattened brainstem. Like her brother, she had a progressive thoracolumbar scoliosis with a Cobb angle of 26°, and had been treated with physiotherapy and corrective brace therapy. Motor development was delayed. In addition to the typical signs of HGPPS, she had bilateral early-onset sensorineural hearing impairment and wore hearing aids.

### Molecular results of the sibling pair patient 1 and 2, their parents and cDNA analysis

A homozygous missense mutation (c.3319A>C) next to the splice donor site of exon 22 of the *ROBO3* gene was found in the two affected siblings (patients 1 and 2). Both parents were heterozygous carriers. Out of 144 controls, one individual of Turkish origin carried the same mutation in a heterozygous state.

**Figure 4:**
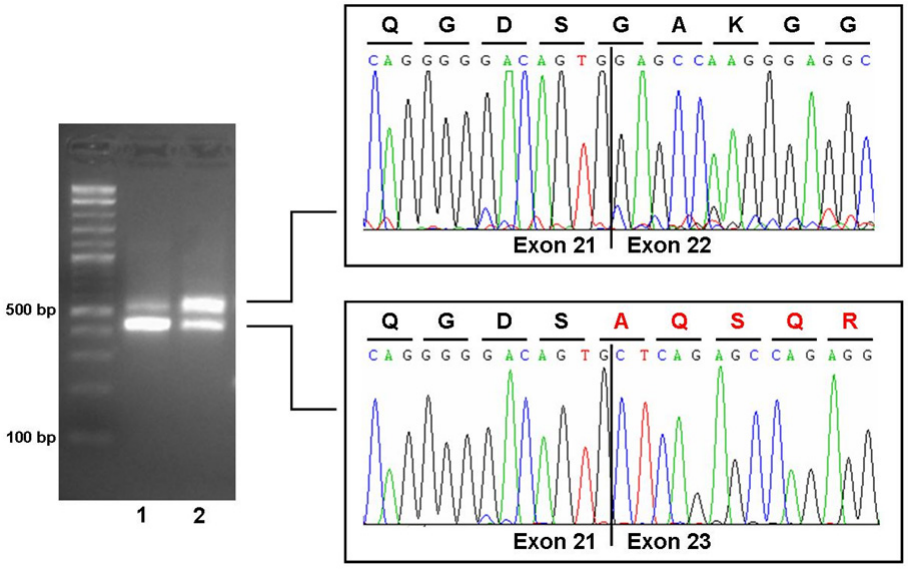
cDNA analysis for c.3319A>C mutation of the *ROBO3* gene. Reverse transcription (RT)-PCR showed leaky splicing with a weak wild-type band with an expected size of 576 bp and a stronger band of around 450 bp, yielded by the skipping of exon 22 of the *ROBO3* gene, for patient 2 (lane 1). For the heterozygous father (lane 2), RT–PCR showed a stronger wild-type band and a weaker band for the exon 22 skipped product. Sequencing of the different products showed unaltered splicing for the larger product (upper electropherogram) and skipping of exon 22 of the *ROBO3* gene (electropherogram below) for the smaller product leading to a frameshift and premature stop codon on the protein level.

The nucleotide exchange was situated 2 bp upstream of the donor splice site of exon 22 of the *ROBO3* gene, and in-silico splice-site analysis predicted a decreased splice efficacy (0.94 for the wild type versus 0.75 for the mutation). cDNA analysis of whole blood confirmed an altered splicing. The mutation led to the skipping of exon 22 of the *ROBO3* gene (Figure 4). On the protein level, the deletion of exon 22 of the *ROBO3* gene most probably results in a frameshift followed by a premature stop codon after 16 altered amino acids.

### Clinical and molecular findings in patient 3

The 13-year-old boy had a progressive right-curved thoracolumbar scoliosis. Due to a rapid progression of scoliosis to a Cobb angle of 52°, corrective surgical intervention was performed using dorsal double-rod instrumentation.

**Figure 5:**
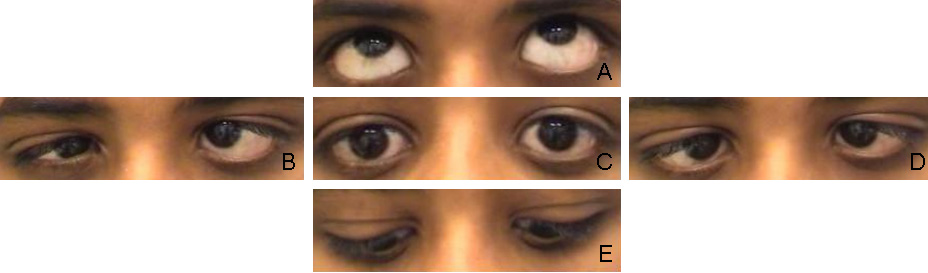
Eye motility in patient 3. The eyes are shown at upgaze (**A**), at right gaze (**B**), in primary position (**C**), left gaze (**D**) and downgaze (**E**).

His horizontal eye movements were severely disturbed: During smooth pursuits at near fixation with a target moving to a side, no abduction occurred, but the eyes converged grossly. Thus, the target was followed by the adducted eye in the manner of a crossed fixation. Vertical movements were normal (Figure 5). No nystagmus was observed. He had left microhypertropia with fusion, as shown under Bagolini’s striated glasses. Corrected visual acuity in anisometropia and astigmatism was 0.8 in the right eye and 0.4 in the left eye with amblyopia in the left eye. Motor development was delayed. The parents from Saudi Arabia were first cousins (Figure 2B). They had unimpaired eye motility.

**Figure 6:**
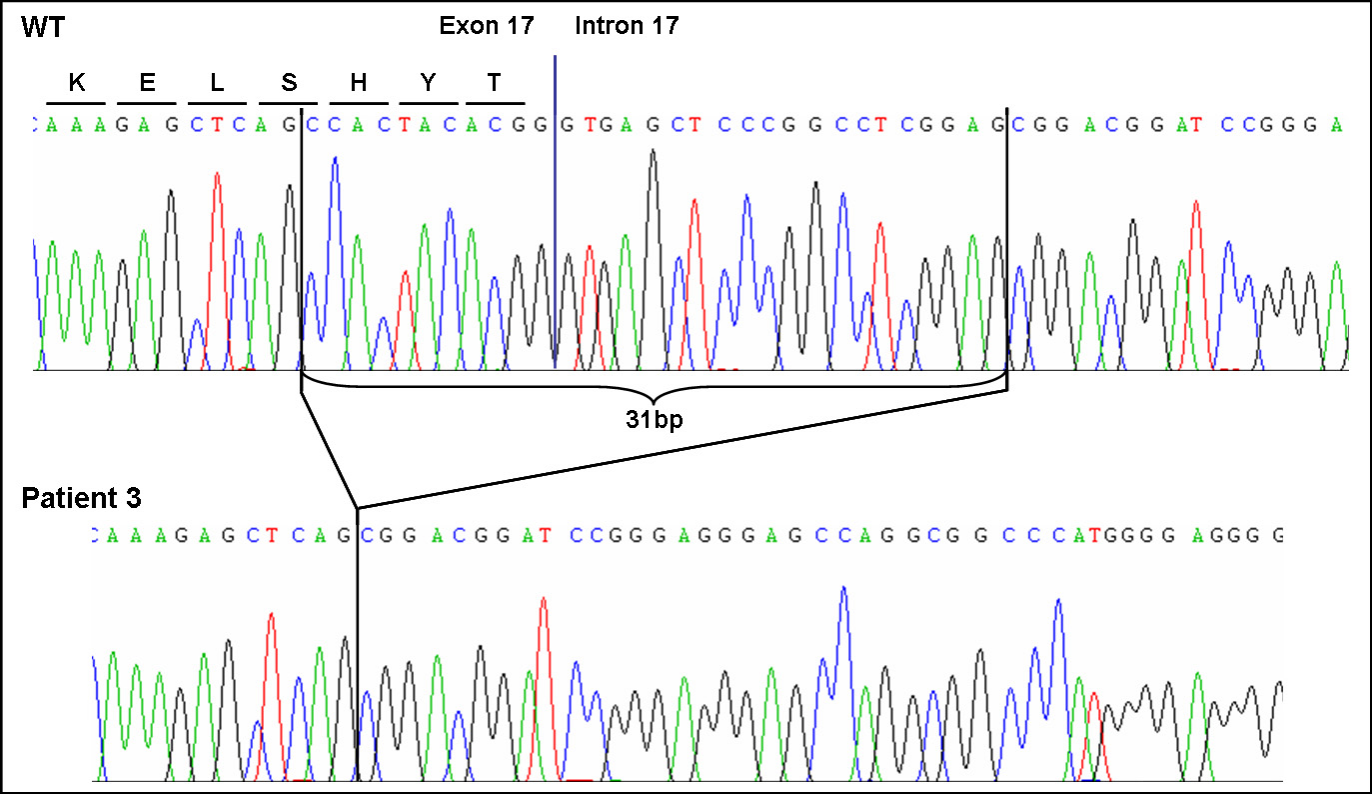
Sequencing chromatogram of the mutation c.2769_2779del11, 2779+1_+20del20 of the *ROBO3* gene. The homozygous deletion of 31 bp of the *ROBO3* gene in patient 3 includes the splice donor site of exon 17 and most probably leads to an altered splicing.

Direct sequencing revealed a homozygous deletion of 31 bp (c.2769_2779del11, 2779+1_+20del20) spanning the splice donor site of exon 17 of the *ROBO3* gene (Figure 6) most probably leading to an altered splicing. Both parents were heterozygous carriers. Neither RNA from the parents nor DNA from the other affected and unaffected siblings was available for molecular testing.

### Clinical and molecular findings in patient 4

The first son of parents of Turkish origin who were first cousins (Figure 2C) was referred at the age of three months because of his head turn to the right and nystagmus, which had intermittently been recognized since he was ten weeks old. Neither parent showed any signs of ocular motility disorders. A female cousin of the patient had both eye motility disorders and spinal column problems, but she had not yet been clinically investigated. At the age of three months, the patient showed deficiency of conjugate horizontal eye movements. Neither horizontal smooth pursuits nor saccades could be elicited, whereas vertical motility seemed unimpaired. A horizontal pendular dissociated nystagmus with larger amplitudes in the left eye existed. Nystagmus became more obvious and seemed to be intermittently converging in reaction to stimuli to elicit an optokinetic response. We saw adducting, but no abducting, movements under the condition of the doll's head maneuver with the right eye adducting when the head was turned to the right and with the left eye adducting when the head was turned to the left. Therefore, both HGPPS and bilateral Duane’s syndrome were considered to be alternative diagnoses.

**Figure 7:**
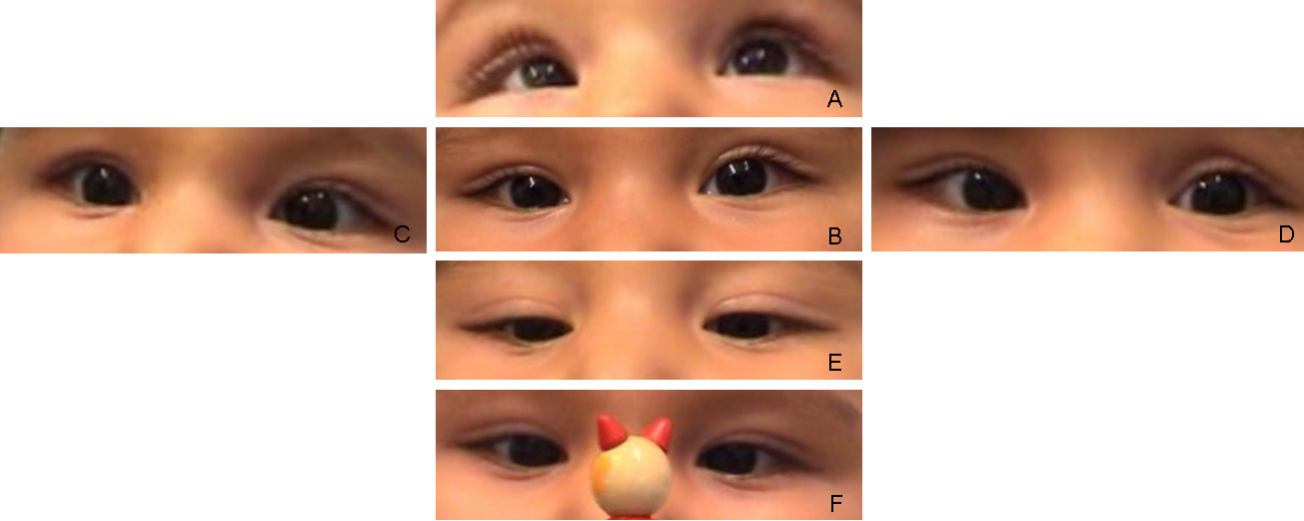
Eye motility in patient 4 at the age of 9 months. Eye position is shown at upgaze (**A**), primary position (**B**), right gaze (**C**), left gaze (**D**), downgaze (**E**), and under the condition of convergence (**F**).

**Figure 8:**
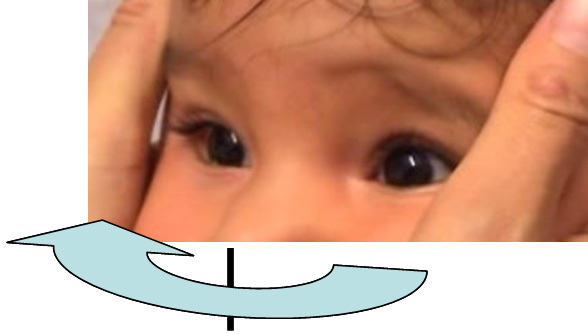
Doll’s head maneuver in patient 4 at the age of 9 months. Under passive rotation of the head to the right compensatory eye movements to the left can not be observed.

**Figure 9:**
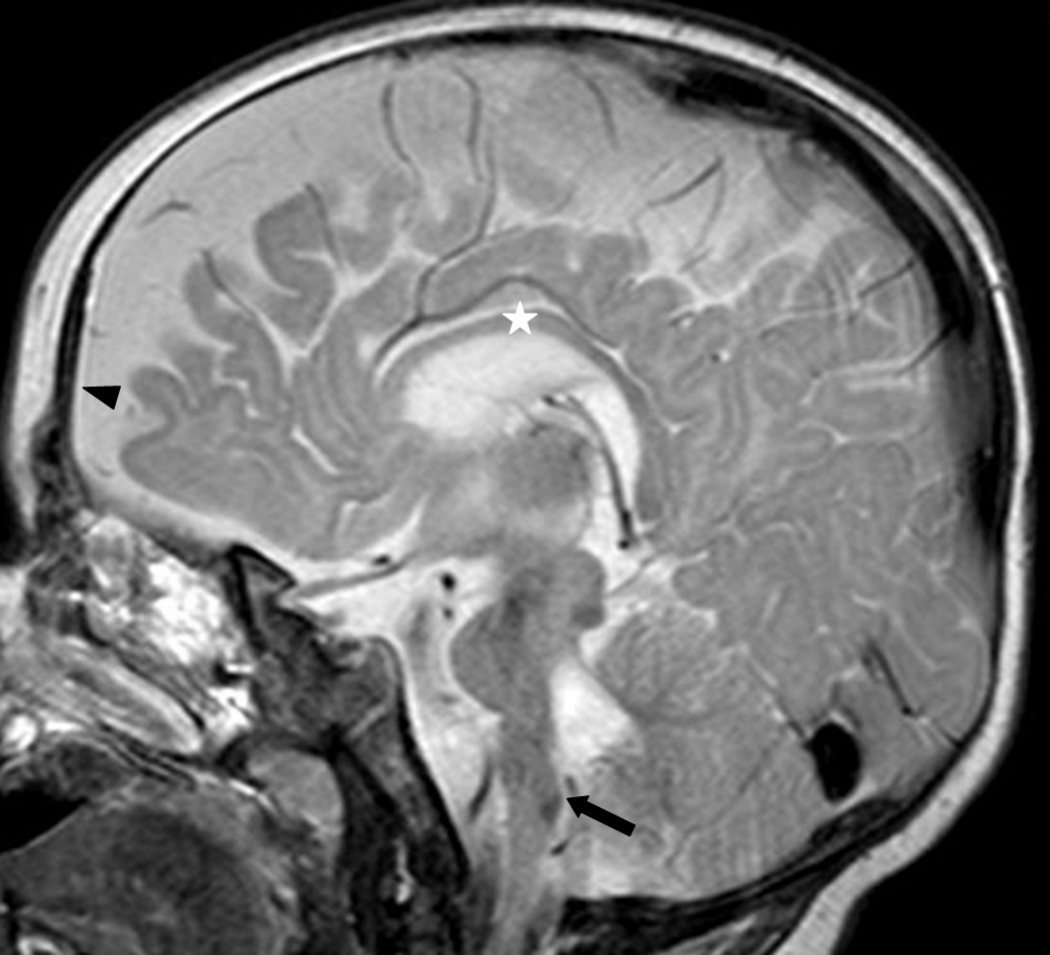
Sagittal magnetic resonance imaging scan of patient 4 at the age of 6 months. The frontal lobes are hypoplastic (arrowhead). The corpus callosum is thinned (star) and the brainstem is flattened (arrow).

Eye position recorded with the Hirschberg test showed −7° of exotropia and slight hypertropia of the left eye. Bilateral slight hyperopia and astigmatism existed. The neuropediatric examination revealed that a posture to the right was preferred. At the ages of nine months and one year, under the condition of smooth pursuit, an adduction of 5° was seen. Horizontal saccades could not be observed. Vestibulo-ocular maneuvers no longer elicited any horizontal movements. The eyes had become aligned. Dissociated pendular nystagmus with larger amplitudes in the left eye persisted. Under conditions of convergence to a near target, the right eye could be adducted 10°, the left eye 17°. By these vergence movements, nystagmus was dampened. Figure 7 and Figure 8 show his eye movements at the age of nine months. MRI at the age of six months shows hypoplasia of the frontal lobes and the corpus callosum (Figure 9). The brainstem and medulla show a malformation presenting with a butterfly configuration in axial scans.

**Figure 10:**
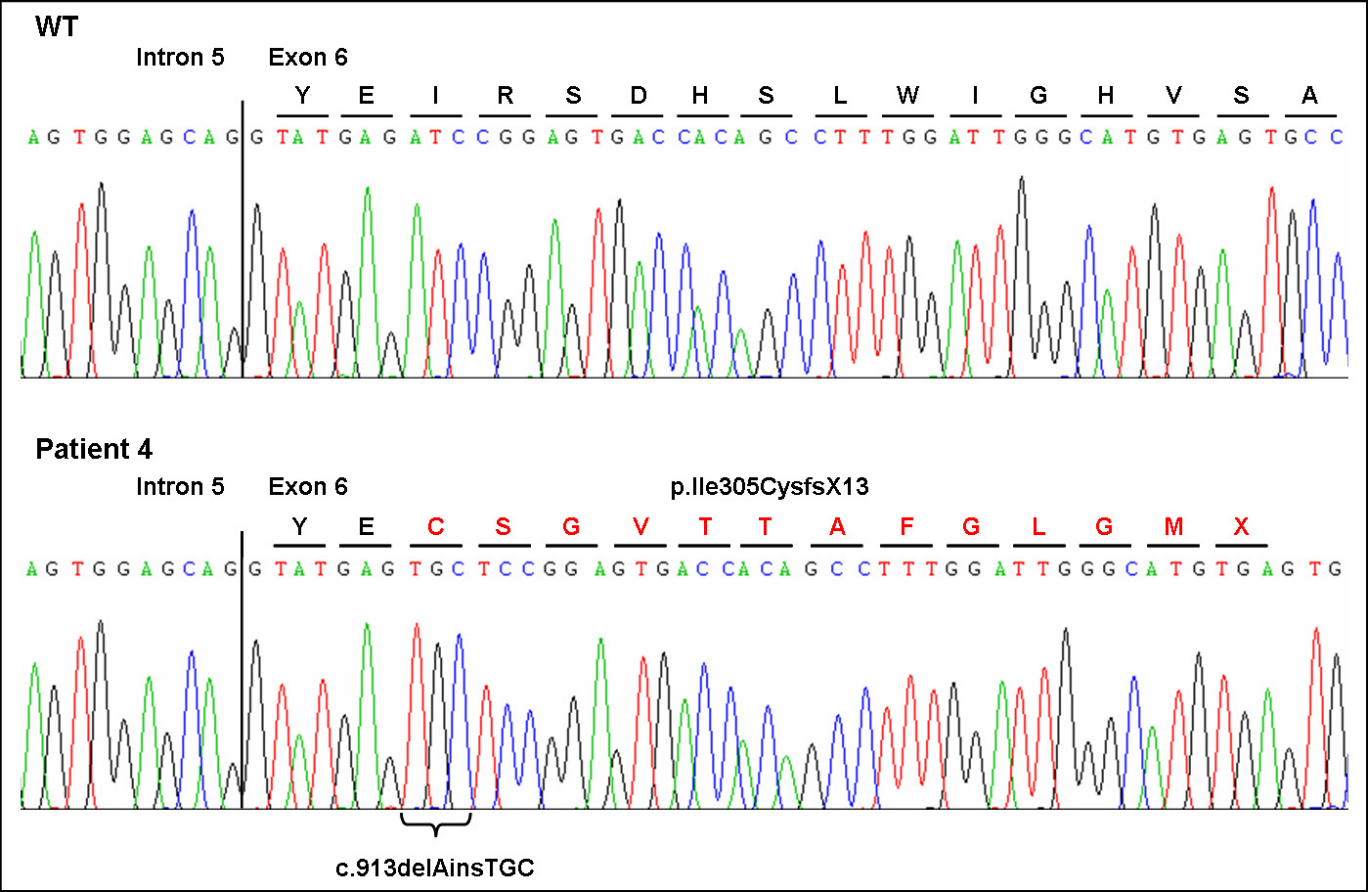
Sequencing chromatogram of the insertion/deletion mutation c.913delAinsTGC of the *ROBO3* gene. The homozygous mutation in patient 4 most likely leads to a frame shift and premature stop codon (p.Ile305CysfsX13).

Molecular findings: A homozygous *ROBO3* insertion/deletion mutation was identified (c.913delAinsTGC; p.Ile305CysfsX13; Figure 10). This frameshift mutation led to a premature stop of translation 12 altered amino acids downstream. Both parents were heterozygous for the mutation. None of the controls showed this mutation.

## Discussion

To date, 24 different mutations affecting the coding region of the *ROBO3* gene have been described. We identified three novel homozygous mutations, that is, a splice-site mutation, the first larger deletion, and an insertion/deletion mutation in the *ROBO3* gene in four HGPPS patients of three consanguineous families from Turkey and Saudi Arabia. Interestingly, the splice-site mutation c.3319A>C was also found in one out of 58 ethnically matched controls. This may point to a founder effect of this mutation or at least to a higher carrier frequency in the Turkish population. Consistent with previous reports, no genotype–phenotype correlation existed but, after comparing the present cases to cases previously reported, some features have to be stressed. Patient 3 showed strong convergence during attempted lateral gaze, which has been previously described in a single patient with HGPPS as synergistic convergence [5]. Scoliosis was present in the three elder patients, whereas the youngest patient did not show any signs of scoliosis at the time of molecular testing, that is, when he was six months old. Since the diagnosis of HGPPS was made before the onset of scoliosis, ongoing orthopedic surveillance is possible and early treatment may help to attenuate its progression.

Remarkably, patient 4 shows signs of widened cerebral anterior fluid spaces. This is a finding only reported once before, in a patient with HGPPS [2]. In our patient, we saw a hypoplastic corpus callosum, which has not been reported in HGPPS before. Based on our finding, special attention should be paid to the corpus callosum in MRI examinations of individuals with a *ROBO3* mutation. Patient 2 had sensorineural hearing loss. No association of early-onset bilateral hearing loss and HGPPS has been reported yet. However, it should be pointed out that, given the parents’ consanguinity, a trait independent from the mutation in *ROBO3* is possible for both the hypoplastic corpus callosum and the sensorineural hearing loss.

Interpreting oculomotor findings in HGPPS remains a challenge and is a field open to speculation. Normally, saccades are generated in the medial pontine reticular formation. The ipsilateral abducent neurons are activated by excitatory neurons, while the contralateral abducent neurons are addressed by inhibitory neurons. Via the abducent internuclear neurons, the information to shift the contralateral eye in the same direction is passed to the contralateral oculomotor nucleus neurons that innervate the medial rectus. Analogously, horizontal vestibular pathway signals are combined at the abducent nucleus level, but the abducent nucleus receives excitatory input from the contralateral side. Along this path, the commands for horizontal smooth pursuits are passed to the abducent nucleus level [11,12]. Assuming that fibers which normally cross the midline might be involved in the defects in HGPPS at the prenuclear level, the crossing inhibitory fibers of the pontine reticular formation and the crossing excitatory fibers that pass signals from the medial vestibular nucleus to the contralateral abducent nucleus may play a role. As in the *ROBO3* mouse model, an overall crossing problem in the hindbrain region occurs [13]—one might presume that the commissural fibers of the abducent internuclear neurons fail to cross the midline.

The failure not only of axons but also of neurons to cross the midline during development may also be a possible pathogenetic mechanism [14]. To explain the pathology of horizontal gaze in HGPPS, it is important to learn both from electrophysiologic and from radiologic studies in patients with HGPPS [8,9], as well as from animal studies on *ROBO3* and its homologs [13,15], that fibers which fail to cross the midline can connect with ipsilateral targets. In HGPPS, both excitatory and inhibitory premotor inputs might reach the abducent nucleus, giving rise to a premotor blockage.

If, by some imbalance, no blockage occurred, but instead an input to the nucleus were passed to the ipsilateral abducent motoneurons and to the internuclear abducent neurons, the result would be a co-contraction of the lateral and medial rectus of the eye on intended abduction, producing a retraction potentially accompanied by narrowing of the lid fissure. Potential termination of the internuclear neurons on the oculomotor neurons of the medial rectus on the side ipsilateral to the abducent nucleus would be the pathophysiological explanation. This could cause a retraction of the globe and explain the narrowing of the lid fissure observed in patient 1.

Moreover, the ascending tract of Deiters may play a role in the findings in one of our patients. We speculate whether the adduction on the horizontal doll’s head maneuver in patient 4 at an early age was mediated by this noncrossing tract, unless the movement seen was an artifact resulting from near convergence. Nevertheless, this reproducible movement led to the initial diagnosis of bilateral Duane’s retraction syndrome in patient 1. Finally, the question as to the mechanism by which the horizontal pendular nystagmus was elicited has to be answered once the mechanism of horizontal gaze palsy in HGPPS is understood. This might also elucidate aspects of the pathophysiology of other forms of nystagmus.

## References

[1] Jen JC, Chan WM, Bosley TM, Wan J, Carr JR, Rub U, Shattuck D, Salamon G, Kudo LC, Ou J, Lin DD, Salih MA, Kansu T, Al Dhalaan H, Al Zayed Z, MacDonald DB, Stigsby B, Plaitakis A, Dretakis EK, Gottlob I, Pieh C, Traboulsi EI, Wang Q, Wang L, Andrews C, Yamada K, Demer JL, Karim S, Alger JR, Geschwind DH, Deller T, Sicotte NL, Nelson SF, Baloh RW, Engle EC (2004). Mutations in a human ROBO gene disrupt hindbrain axon pathway crossing and morphogenesis.. Science.

[2] Chan WM, Traboulsi EI, Arthur B, Friedman N, Andrews C, Engle EC (2006). Horizontal gaze palsy with progressive scoliosis can result from compound heterozygous mutations in ROBO3.. J Med Genet.

[3] Amouri R, Nehdi H, Bouhlal Y, Kefi M, Larnaout A, Hentati F (2009). Allelic ROBO3 heterogeneity in Tunisian patients with horizontal gaze palsy with progressive scoliosis.. J Mol Neurosci.

[4] Abu-Amero KK, al Dhalaan H, al Zayed Z, Hellani A, Bosley TM (2009). Five new consanguineous families with horizontal gaze palsy and progressive scoliosis and novel ROBO3 mutations.. J Neurol Sci.

[5] Khan AO, Oystreck DT, Al-Tassan N, Al-Sharif L, Bosley TM (2008). Bilateral synergistic convergence associated with homozygous ROB03 mutation (p.Pro771Leu).. Ophthalmology.

[6] Ng AS, Sitoh YY, Zhao Y, Teng EW, Tan EK, Tan LC (2011). Ipsilateral Stroke in a Patient With Horizontal Gaze Palsy With Progressive Scoliosis and a Subcortical Infarct.. Stroke.

[7] Bosley TM, Salih MA, Jen JC, Lin DD, Oystreck D, Abu-Amero KK, MacDonald DB, al Zayed Z, al Dhalaan H, Kansu T, Stigsby B, Baloh RW (2005). Neurologic features of horizontal gaze palsy and progressive scoliosis with mutations in ROBO3.. Neurology.

[8] Haller S, Wetzel SG, Lutschg J (2008). Functional MRI, DTI and neurophysiology in horizontal gaze palsy with progressive scoliosis.. Neuroradiology.

[9] Amoiridis G, Tzagournissakis M, Christodoulou P, Karampekios S, Latsoudis H, Panou T, Simos P, Plaitakis A (2006). Patients with horizontal gaze palsy and progressive scoliosis due to ROBO3 E319K mutation have both uncrossed and crossed central nervous system pathways and perform normally on neuropsychological testing.. J Neurol Neurosurg Psychiatry.

[10] Sicotte NL, Salamon G, Shattuck DW, Hageman N, Rub U, Salamon N, Drain AE, Demer JL, Engle EC, Alger JR, Baloh RW, Deller T, Jen JC (2006). Diffusion tensor MRI shows abnormal brainstem crossing fibers associated with ROBO3 mutations.. Neurology.

[11] Büttner U, Büttner-Ennever JA (2006). Present concepts of oculomotor organization.. Prog Brain Res.

[12] Leigh RJ, Zee DS. The neurology of eye movements. 4 ed. New York: Oxford Univ. Press; 2006.

[13] Sabatier C, Plump AS, Le M, Brose K, Tamada A, Murakami F, Lee EY, Tessier-Lavigne M (2004). The divergent Robo family protein rig-1/Robo3 is a negative regulator of slit responsiveness required for midline crossing by commissural axons.. Cell.

[14] Marillat V, Sabatier C, Failli V, Matsunaga E, Sotelo C, Tessier-Lavigne M, Chedotal A (2004). The slit receptor Rig-1/Robo3 controls midline crossing by hindbrain precerebellar neurons and axons.. Neuron.

[15] Burgess HA, Johnson SL, Granato M (2009). Unidirectional startle responses and disrupted left-right co-ordination of motor behaviors in robo3 mutant zebrafish.. Genes Brain Behav.

